# Flood-Associated, Land-to-Sea Pathogens’ Transfer: A One Health Perspective

**DOI:** 10.3390/pathogens12111348

**Published:** 2023-11-14

**Authors:** Giovanni Di Guardo

**Affiliations:** General Pathology and Veterinary Pathophysiology, Veterinary Medical Faculty, University of Teramo, Località Piano d’Accio, 64100 Teramo, Italy; gdiguardo@unite.it

Similarly to many other countries across the globe, several floods have been recorded in Italy throughout the last few decades, including those of catastrophic magnitude that occurred in the Emilia-Romagna and Tuscany regions last May and a few weeks ago, respectively. This, once again, underscores the increasingly growing probability, in the current “*Anthropocene Epoch*”, of global warming-related, extreme weather phenomena. Indeed, the last 8 years (2015–2022) have been characterized by the highest average temperatures ever recorded on Earth throughout the last 140 years [1].

“*How can we imagine to stay healthy in a sick world?*”, Pope Francis wrote three years ago in his missive addressed to the President of Columbia on the “*2020 World Environment Day*”, while the COVID-19 pandemic was dramatically affecting the entire world, with SARS-CoV-2 likely representing a clear-cut example of a climate change-driven pathogen spillover from bats to humans [2].

Within such a challenging and alarming scenario, the land-to-sea transfer of a huge (and progressively increasing) number of infectious agents appears to be a matter of relevant concern [3,4]. This especially applies to bacterial microorganisms shed into the external environment via the fecal route, such as *Salmonella* spp., *Escherichia coli*, *Vibrio cholerae* and *Listeria monocytogenes*, alongside protozoan pathogens like *Toxoplasma gondii* and/or viral agents like the one causing hepatitis A and, last but not least, the pandemic SARS-CoV-2 betacoronavirus. As a matter of fact, evidence of viral fecal shedding has been documented for a median duration of 22 days in 59% of subjects from a cohort of SARS-CoV-2-infected patients in China [5]. Once transferred into sea and ocean waters by flood-derived mud and debris, fecally excreted microbial pathogens may be ingested by edible bivalve mollusks like mussels, an organism in which a single individual is able to filter over 100 liters of water on a daily basis, thus potentially hosting inside its body tissues significant amounts of biological and chemical environmental pollutants [6]. Within this context, it is worth mentioning a *V. cholerae* infection outbreak linked to the consumption of raw, non-sterilized mussels which diffusely involved the human population from the cities of Naples and Bari during the summer and early autumn months of 1973 [7]. Moreover, the land-to-sea transfer of infectious agents may additionally involve free-ranging cetaceans, whose health and conservation *status* appear to be increasingly threatened by a long and progressively expanding list of both natural and anthropogenic factors. This holds particularly true for “inshore” species like bottlenose dolphins (*Tursiops truncatus*), which are more prone to acquire infections caused by “terrestrial” pathogens like *T. gondii* [8] while being simultaneously able to accumulate and “biomagnify” inside their body tissues consistent amounts of human-made, persistent, immunotoxic, neurotoxic and endocrine-disrupting environmental pollutants, based upon their well-known position of “apex predators” within the marine and oceanic food chains. Furthermore, the proven capability of micro-nanoplastics—exceedingly contaminating global seawaters—to behave as “attractors and concentrators” for the aforementioned anthropogenic xenobiotics should also be taken into serious account, together with the demonstrated interaction of micro-nanoplastics in marine and oceanic ecosystems with zoonotic protozoan pathogens like *T. gondii*, *Cryptosporidium parvum* and *Giardia enterica* [9]. This scenario, which already appears to be quite intricate and complex by itself, is made even more alarming by the fact that micro-nanoplastics may also host and carry a wide range of antibiotic-resistant bacteria, from which an active and powerful exchange of antimicrobial resistance genes may additionally occur, through horizontal gene transfer, with several environmental bacterial species colonizing the same plastic substrates [10].

Interestingly enough, given the significant homology degree of their angiotensin-converting enzyme-2 (ACE-2) viral receptor with the human one, bottlenose dolphins and other aquatic mammal species living in the Mediterranean Sea have been deemed potentially susceptible to SARS-CoV-2 infection [11], which to the best of my knowledge has not been hitherto reported, however, in any cetacean species. In this respect, performing extensive sero-epidemiological investigations against SARS-CoV-2 in potentially susceptible cetaceans (and pinnipeds) under human care kept inside aquaria, delphinaria and zoological gardens should be regarded as a priority, given their repeated close contacts both with trainers and with the general public, coupled with the documented occurrence of SARS-CoV-2 infection in several free-living and captive animal species [12].

Based upon the concepts and data presented above, in parallel with the extremely urgent and no more postponable need to invest adequate money sums into what could be tentatively called an “*extreme weather event-related preparedness and readiness operational machine*”, I would also like to emphasize herein that climate change, global warming and climate change-related health issues should be opposed by adopting an ad hoc “paradigm shift” and through a multidisciplinary, transnational and transcontinental, basic and applied research effort, constantly inspired by the “*One Health, One Earth, One Ocean*” perspective, which reminds us that human, animal and environmental health are inextricably and reciprocally linked to each other!
